# Locked Nucleic Acid Probe-Based Real-Time PCR Assay for the Rapid Detection of Rifampin-Resistant *Mycobacterium tuberculosis*

**DOI:** 10.1371/journal.pone.0143444

**Published:** 2015-11-24

**Authors:** Yong Zhao, Guilian Li, Chongyun Sun, Chao Li, Xiaochen Wang, Haican Liu, Pingping Zhang, Xiuqin Zhao, Xinrui Wang, Yi Jiang, Ruifu Yang, Kanglin Wan, Lei Zhou

**Affiliations:** 1 Laboratory of Analytical Microbiology, State Key Laboratory of Pathogen and Biosecurity, Beijing Institute of Microbiology and Epidemiology, Beijing 100071, P. R. China; 2 Beijing Key Laboratory of POCT for Bioemergency and Clinic (No. BZ0329), Beijing 100071, P. R. China; 3 State Key Laboratory for Infectious Diseases Prevention and Control, Collaborative Innovation Center for Diagnosis and Treatment of Infectious Diseases, National Institute for Communicable Disease Control and Prevention, Chinese Center for Disease Control and Prevention, Beijing 102206, P. R. China; 4 Department of Clinical Laboratory, Chinese People’s Liberation Army General Hospital, Beijing 100853, P. R. China; 5 College of Animal Science and Technology, Jilin Agricultural University, Changchun 130118, P. R. China; 6 Institute for Plague Prevention and Control of Hebei Province, Zhangjiakou 075000, P. R. China; Cornell University, UNITED STATES

## Abstract

Drug-resistant *Mycobacterium tuberculosis* can be rapidly diagnosed through nucleic acid amplification techniques by analyzing the variations in the associated gene sequences. In the present study, a locked nucleic acid (LNA) probe-based real-time PCR assay was developed to identify the mutations in the *rpoB* gene associated with rifampin (RFP) resistance in *M*. *tuberculosis*. Six LNA probes with the discrimination capability of one-base mismatch were designed to monitor the 23 most frequent *rpoB* mutations. The target mutations were identified using the probes in a “probe dropout” manner (quantification cycle = 0); thus, the proposed technique exhibited superiority in mutation detection. The LNA probe-based real-time PCR assay was developed in a two-tube format with three LNA probes and one internal amplification control probe in each tube. The assay showed excellent specificity to *M*. *tuberculosis* with or without RFP resistance by evaluating 12 strains of common non-tuberculosis mycobacteria. The limit of detection of *M*. *tuberculosis* was 10 genomic equivalents (GE)/reaction by further introducing a nested PCR method. In a blind validation of 154 clinical mycobacterium isolates, 142/142 (100%) were correctly detected through the assay. Of these isolates, 88/88 (100%) were determined as RFP susceptible and 52/54 (96.3%) were characterized as RFP resistant. Two unrecognized RFP-resistant strains were sequenced and were found to contain mutations outside the range of the 23 mutation targets. In conclusion, this study established a sensitive, accurate, and low-cost LNA probe-based assay suitable for a four-multiplexing real-time PCR instrument. The proposed method can be used to diagnose RFP-resistant tuberculosis in clinical laboratories.

## Introduction

The tuberculosis caused by drug-resistant strains of *Mycobacterium tuberculosis* has been considered as one of the most critical public health issues. The early diagnosis of drug-resistant tuberculosis via a rapid and reliable method can facilitate the administration of effective anti-tuberculosis drugs to patients and prevent the further spread of drug-resistant strains [1]. However, conventional culture-based drug susceptibility test requires several weeks to identify the cases of drug-resistant tuberculosis because of the slow growth of *M*. *tuberculosis* [2]. Over the last two decades, studies have considerably focused on the development of new and rapid methods that can be used to diagnose drug-resistant *M*. *tuberculosis* in a short period [3–5].

Nucleic acid amplification technique (NAAT) is currently one of the most rapid and sensitive molecular diagnostic tools to detect drug-resistant tuberculosis, which is mechanically developed on the basis of the genetic variations associated with drug resistance in *M*. *tuberculosis* [6, 7]. Though the genetic basis of drug resistance is only partially understood, several genes, such as *rpoB*, *inhA*, *KatG*, *gyrA*, and *gyrB*, in *M*. *tuberculosis* genome have been well documented because they have been highly implicated in drug resistance [8]. In particular, 95% of rifampin (RFP)-resistant strains contains mutations localized in the *rpoB* gene that encodes the β-subunit of the bacterial RNA polymerase. Reported mutations associated with RFP-resistance in the gene were accumulated in the 81-bp (codon 507 to codon 533) region called the rifampin resistance determination region (RRDR) [8–10]. Moreover, the most high-frequency mutations were localized at codon 516, 526, and 531, as observed worldwide [9, 11, 12]. Consequently, the *rpoB* genotype of *M*. *tuberculosis* has been considered as a genetic target to detect RFP resistance through NAATs.

Among the currently used NAATs, probe-based real-time PCR is one of the most prevalent and reliable approaches to detect drug-resistant *M*. *tuberculosis*. This approach can detect gene mutations associated with drug resistance by using the oligonucleotide probe, which is characteristic of mutation discrimination capability. Such probes used in the previously reported assays include the molecular beacon [13, 14], the TaqMan minor groove binder (MGB) probe [15], the sloppy molecular beacon[10], the dual-labeled probe[16, 17], and the padlock probe[18] and others. However, none of these probes exhibit rigid specificity to discriminate single-base mutations in the RRDR of *M*. *tuberculosis* in a simple manner. The molecular beacon-based and Taqman MGB-based assays discriminated the wild-type sequences from the mutations based on their differences of quantification cycles (ΔCq) [14, 15]. The sloppy molecular beacon-based and dual-labelled probe-based approaches need additional Tm analyses after the amplification[19]. Padlock probes were used in the rolling circle amplifications, which required complex procedure [18]. In addition to above probes, LNA probe is another kind of probe that has been widely used in real-time PCR to quantitatively detect pathogens [20, 21]. The LNA probe contains oligonucleotide monomers modified with an additional methylene bridge between 2′ oxygen and 4′ carbon of the ribose ring. The modified LNA monomer or monomers in the probe can increase the thermal stability of the probe and help develop short probe designs, which can improve capability of the probe to detect mutations [22]. The reported advantages of the probes include superior sensitivity and specificity to detect mutation, ease of design, and preferable signal-to-noise ratio [23, 24].

In the study, a LNA-probe real-time PCR assay was developed to detect RFP-resistant strains of *M*. *tuberculosis*. Six LNA probes that can discriminate one-base mutations have been designed to identify mutations in the RRDR of the *rpoB*. According to the reports [14], a total of 23 common *rpoB* mutations associated with RFP-resistant *M*. *tuberculosis*, including high-frequency mutations at codon 516, 526, and 531, were collected as the targets to evaluate the LNA probe-based assay. The LNA probe is highly specific; thus, any one-base mismatch can be easily identified through the amplification curves (Cq = 0), without complex determinations or additional Tm analyses. And the proposed assay showed sufficient specificity and sensitivity to detect *M*. *tuberculosis* and RFP-resistant *M*. *tuberculosis*, validated by a blind test of 154 clinical isolates.

## Materials and Methods

### Clinical isolates

A reference strain of *M*. *tuberculosis* H37Rv (ATCC 27294) and 142 clinical isolates of *M*. *tuberculosis* with well-defined information on drug susceptibility and *rpoB* gene sequence were collected for the present study. The isolates were preserved and cultured at the State Key Laboratory for Infectious Disease Prevention and Control, Collaborative Innovation Center for Diagnosis and Treatment of Infectious Diseases, National Institute for Communicable Disease Control and Prevention, and Chinese Center for Disease Control and Prevention (Beijing, P. R. China). The 142 isolates of *M*. *tuberculosis* were determined with 88 RFP-susceptible strains and 54 RFP-resistant strains. DNA sequences of the RFP-resistant strains contains 17 types of *rpoB* mutations (S1 Table), covering the most frequent mutations at codons 516, 526, and 531. The RFP-susceptible strains showed no mutations in the RRDR region of the *rpoB* gene.

Another set of 12 clinical isolates of non-tuberculosis mycobacterium (NTM) were also collected in the study, including *Mycobacterium smegmatis*, *M*. *avium*, *M*. *terrae*, *M*. *shimodii*, *M*. *kansasii*, *M*. *asiaticum*, *M*. *scrofulaceum*, *M*. *gordanea*, *M*. *chelonea*, *M*. *abscessus*, *M*. *fortuitum*, *and M*. *phlei*.

### Cloning of the *rpoB* gene

Corresponding to the *rpoB* sequences from the *M*. *tuberculosis* H37Rv genome (GenBank accession no. L27989) and the 23 common mutations associated with RFP resistance (Table 1), *rpoB* segments (280 bp) containing the RRDR of wild and mutant types were synthesized and cloned into the pUC 57 vector. The plasmid DNA was used to initially evaluate and optimize probe specificity.

**Table 1:** Summary of the assay performance for the detection of *rpoB* mutations.

Mutation codon	Mutation type	Cq values					
		LNA-P1	LNA-P2	LNA-P3	LNA-P4	LNA-P5	LNA-P6
**507** ^#^	**Deletion**	N*	27.78	27.84	28.07	29.52	28.43
**510**	**CAG-CAT**	24.08	N	24.43	22.96	24.43	25.95
**511**	**CTG-CCG**	26.81	N	26.91	28.17	29.46	28.66
**511** ^#^	**CTG-CGG**	26.97	N	27.3	26.15	27.57	28.51
**513**	**CAA-AAA**	26.27	N	26.61	27.58	29.32	27.98
**513** ^#^	**CAA-CCA**	27.51	N	27.83	26.61	28.03	29.46
**513** ^#^	**CAA-CTA**	23.69	N	24.00	22.83	24.19	25.60
**516**	**GAC-TAC**	24.47	27.62	N	27.70	29.82	26.41
**516**	**GAC-GTC**	23.49	27.18	N	27.27	29.53	25.27
**516**	**GAC-GGC**	27.91	31.68	N	32.91	33.87	29.06
**516–517** ^#^	**Deletion**	22.15	23.85	N	24.11	25.42	24.15
**522**	**TCG-TTC**	23.51	20.82	23.83	N	21.36	25.27
**522** ^#^	**TCG-CAG**	29.03	25.99	29.35	N	26.32	30.73
**522** ^#^	**TCG-TGG**	26.84	24.27	27.14	N	24.60	28.03
**526**	**CAC-TAC**	26.74	28.3	27.06	28.38	N	28.43
**526**	**CAC-AAC**	25.85	25.32	26.2	25.53	N	27.45
**526**	**CAC-GAC**	26.07	27.21	26.58	27.32	N	27.33
**526**	**CAC-TGC**	24.84	26.26	25.24	26.38	N	26.35
**526** ^#^	**CAC-CCC**	23.01	22.65	23.40	23.15	N	26.49
**526**	**CAC-CGC**	26.98	29.29	27.03	29.95	N	28.71
**526**	**CAC-CTC**	25.49	26.91	25.83	26.97	N	27.03
**531**	**TCG-TTG**	27.59	28.27	27.59	29.01	29.80	N
**531**	**TCG-TTT**	23.95	26.14	24.35	26.20	26.63	N

^#^ Plasmid DNA template;

* Negative result.

### DNA extraction

Chromosomal DNA was extracted from fresh bacterial cultures via traditional cetyltrimethylammonium bromide (CTAB) method [25]. In brief, the bacterial cells were digested with lysozyme and proteinase K in the presence of sodium dodecyl sulfate. DNA was released from proteins and other components by using CTAB (final concentration, 40 mM) and then extracted using chloroform-isoamyl alcohol (24:1). The genomic DNA was then isolated through ethanol precipitation and resuspended in Tris-EDTA buffer for storage.

Plasmid DNA was extracted using a QIAamp DNA mini kit (Qiagen, Hilden, Germany) in accordance with the manufacturer’s instructions. The DNA samples were stored at −20°C.

### Primers and LNA probes preparation

The real-time PCR primers and probes used in the study are shown in S2 Table. The primers used to amplify *rpoB* were manually designed to selectively amplify a 206 bp amplicon containing the RRDR. Six LNA probes were designed to identify any mutation in the amplicon and to specifically hybridize with the entire 81 bp RRDR in the wild-type strains by using Beacon Designer software 7.9 (PREMIER Biosoft). Each LNA probe was covalently labeled at the 5′-end with a unique fluorescent reporter dye [6-carboxyfluorescein (FAM), 4,4,7,2′,4′,5′,7′-hexachloro-6-carboxyfluorescein (HEX), or Cy5] and a BHQ at the 3′-end. All of the probes and primers were synthesized by Huirui (Shanghai, China).

The specific primers and probes of *Bacillus globigii* (ATCC 9372) were also used for the internal amplification control (IAC) and were amplified with *M*. *tuberculosis* in one real-time PCR to detect PCR inhibitors. The oligonucleotide sequences of the primers and a Taqman probe were designed using Primer Express 3.0 (Applied Biosystems, Foster City, CA) to target a conserved segment of *BgSP* in *B*. *globigii* (GenBank accession no. L27989). The Taqman probe was labeled with carboxy-X-rhodamine (ROX) to be distinguished from the other dyes.

### Evaluation of LNA probes by plasmid DNA through monoplex-probe real-time PCR

Plasmid DNA templates, including the wild-type template and the corresponding mutant templates, were used to determine the specificity of each LNA probes through a monoplex-probe real-time PCR amplification. The monoplex-probe real-time PCR was performed in a final reaction volume of 25 μl containing 2× LightCycler 480-probe master mix (Roche Diagnostics), forward primer of *rpoB* (*rpoB*-F2, 400 nM), reverse primer of *rpoB* (*rpoB*-R, 400 nM), detecting LNA-probe (240 nM), and 2.5 μl of plasmid DNA template (3.0 × 10^5^ copies/μl). The final volume was adjusted with distilled water. The reaction was run under the following conditions: 95°C for 5 min, followed by 45 cycles of denaturation at 95°C for 15 s, annealing at 65°C for 30 s, and a cooling down step at 37°C for 1 min.

### Establishment of the multiplex-probe real-time PCR assay

Multiplex-probe real-time PCR was run in a two-tube formation (Tube A and Tube B), with three differently labeled LNA-probes and an IAC-probe in each tube. A final reaction volume of 30 μl was used in each tube containing 2× LightCycler 480-probe master mix, primers for *rpoB* (*rpoB*-F2 and *rpoB*-R, 800 nM), primers for *BgSP* (*BgSP*-F and *BgSP*-R, 500 nM), a mixture of probes (LNA-P6, LNA-P3, LNA-P1, and IAC-probe in Tube A, 240 nM), or a mixture (LNA-P2, LNA-P5, LNA-P4, and IAC-probe in Tube B, 240 nM), 2.5 μl of the purified DNA of *B*. *globigii* (1000 GE/μl), and 2.5 μl of the genomic DNA extracted from mycobacteria.

Real-time PCR was conducted using a LightCycler 480 II instrument. The conditions of PCR amplification were as follows: initial activation at 95°C for 5 min, followed by 45 cycles of denaturation at 95°C for 15 s, annealing at 65°C for 30 s, and a cooling down step at 37°C for 1 min. Four filter combinations (excitation spectrum–emission spectrum) were chosen and set as follows: FAM channel (465 nm–510 nm), HEX channel (498 nm–580 nm), Rox channel (533 nm–610 nm), and Cy5 channel (618 nm–660 nm). The fluorescence in the four channels was determined simultaneously during the annealing step. Before fluorescence was analyzed, color compensation was established in accordance with the manufacturer’s instructions. The quantification cycle (Cq) was obtained by using the LightCycler software program (version 4.0) with the second derivative algorithms.

### Result interpretations through the real-time PCR assay

The real-time PCR assay results are described as follows: (1) *M*. *tuberculosis* was determined by positive signals from at least four LNA probes; (2) RFP-susceptible *M*. *tuberculosis* was determined by positive signals from all of the six LNA probes; (3) RFP-resistant *M*. *tuberculosis* was determined by one or two absent probe signals. (4) *M*. *tuberculosis* was precluded if signals were absent in three or more probes. (5) Diagnoses were based on a positive signal from the IAC probe (34 < Cq < 40); otherwise, the test was considered invalid.

### Determination of the assay sensitivity

The genomic DNA of *M*. *tuberculosis* H37Rv was prepared in serial dilutions from 4,000,000 GE/μl to 4 GE/μl to evaluate the assay sensitivity. Then, 2.5 μl of the diluted DNA was used as the template to determine sensitivity for the multiple-probe-based real-time PCR. The templates are tested in triplets at least, as described above. To achieve an improved sensitivity, the nested PCR was introduced into the assay. The first round of the nested PCR was performed in a 20 μl reaction volume containing 2× Light Cycler 480-probe master mix, 250 nM of each primer (*rpoB*-F1, *rpoB*-R), 3 μl of DNA, and distilled water. The reaction was run under the following conditions: 95°C for 5 min, followed by 30 cycles of 10 s at 95°C, 15 s at 60°C, and another 15 s at 72°C. The product from the first round of PCR was diluted 100 times with distilled water and used as the template for the second round of real-time PCR as mentioned above.

### Determination of the assay specificity to *rpoB* mutations associated with RFP resistance

DNA samples with 23 different RRDR sequences were prepared to determine the assay capability to discriminate *rpoB* mutations associated with RFP resistance. Of the templates, 15 were from the genomic DNA of RFP-resistant strains of *M*. *tuberculosis* among the sequenced clinical isolates; the other 8 DNA templates were from the plasmid DNA prepared in the study. All the DNA templates were diluted to the concentration ranged from 10^5^ to 10^6^ GE/tube or copies/tube.

### Determination of the assay specificity by DNA samples from the NTMs

12 NTM strains were collected and used to confirm the detecting specificity of the proposed assay. Genomic DNA samples were extracted from the NTMs as the templates (approximately 0.1 ng/μl) of the LNA-probe based real-time PCR. DNA sequences from drug-susceptible *M*. *tuberculosis* and drug-resistant *M*. *tuberculosis* were also prepared and tested as the control.

### Validation of the assay by clinical isolates

A total of 154 bacteria samples, including 12 NTM strains and 142 clinical isolates with well-defined information on drug susceptibility and *rpoB* sequence, were used to evaluate performance of the assay. Each DNA sample was diluted to the concentration of 10^5^ or 10^6^ GE/tube as the template of the real-time PCR. The DNA samples were coded independently and randomly and tested by the assay in a blind test way.

## Results

### Establishment of the real-time PCR assay

**Fig 1:**
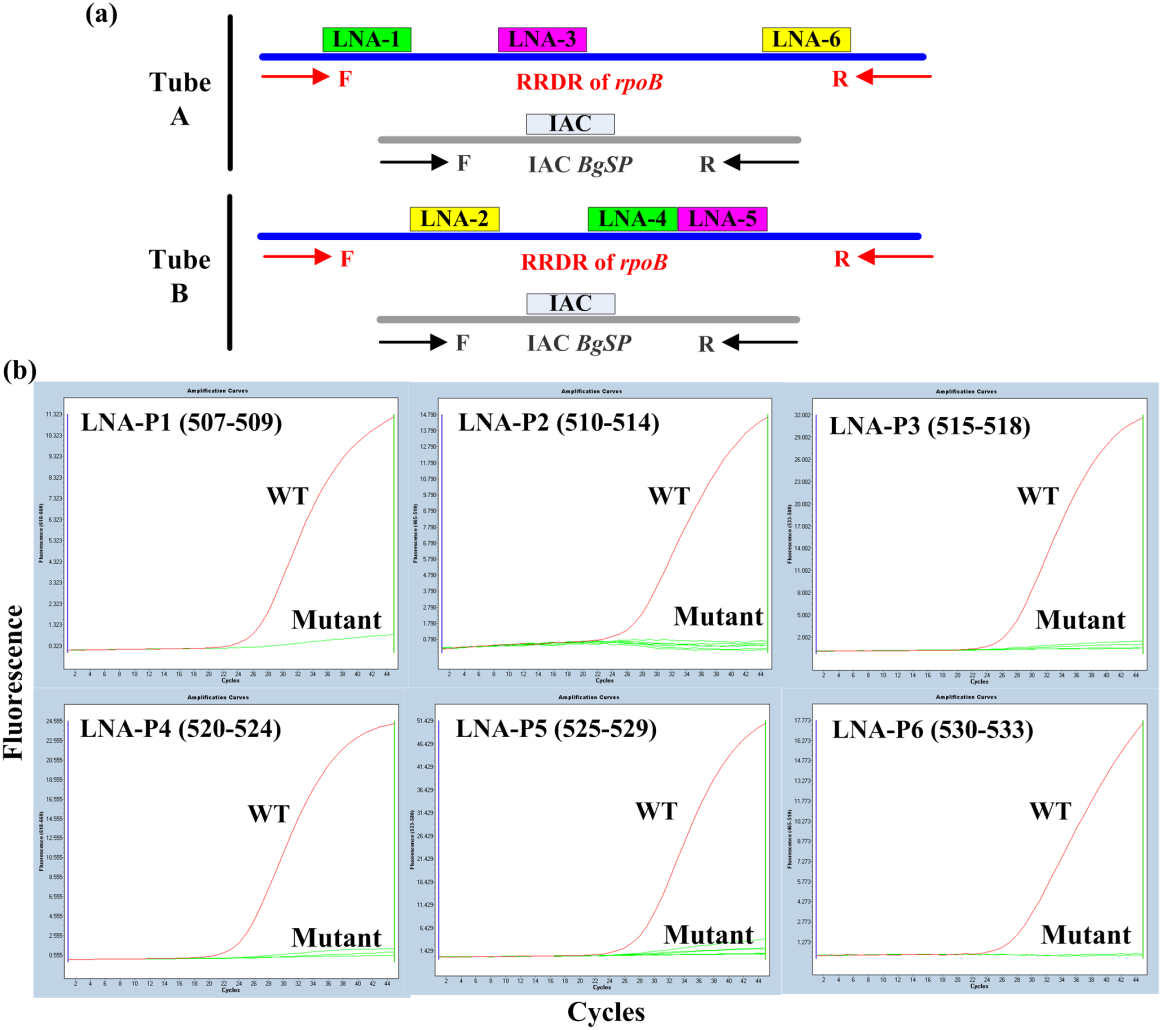
Schematic description of the assay and mutation discrimination capability of the six LNA probes. (a) The assay is performed in a two-tube form with three LNA probes and one IAC probe in each tube to detect *M*. *tuberculosis* and RFP-resistant *M*. *tuberculosis*. The two tubes share the same real-time PCR components except the included probes. (b) The LNA probes used in the assay could discriminate single-base mutations. The single-base variation in the target sequence prevents the corresponding probe matching and combining with the target; as a result, probe dropout from the sequence occurs (Cq = 0).

As presented in the design scheme in Fig 1, six fluorescent LNA probes (LNA-P1 to LNA-P6) were designed to identify the genetic variations in the *rpoB* gene of *M*. *tuberculosis*. The probes were assigned to monitor the RRDR region (from codon 507 to codon 533 of *rpoB*). The LNA probes could identify these single-base mutations in a manner of “probe dropout” (Cq = 0); in this way, the probe could not combine with the mutant target; as a result, negative fluorescence signals were produced during amplification. The six LNA probes were allocated in two tubes to consist of a two-tube form real-time PCR assay. Each tube contained three LNA probes labeled with different dyes. The two reactions were performed simultaneously and shared the same real-time PCR components except the included probes to detect drug-resistant *M*. *tuberculosis*. Probe allocation was determined by comparing different probe combinations (S3 Table). Finally, LNA-P1, LNA-P3, and LNA-P6 were placed in tube A, and LNA-P2, LNA-P4, and LNA-P5 were placed in tube B.

An internal amplification control (IAC) based on *B*. *globigii* (ATCC 9372) was developed to monitor PCR inhibitors in each tube. The IAC system consists of specific primers and a Taqman probe labeled with Rox, which were targeted at a conserved segment in *BgSP* of *B*. *globigii*. Considering the amplification competition between *rpoB* and *BgSP*, we pre-added approximately 2500 GE/tube *B*. *globigii* to the reaction mixture to generate a sufficient IAC signal and significantly reduce the interference with *rpoB* amplification.

### Evaluation of the LNA probe by plasmid DNA

Before the assay with multiple probes was established, the capability of each LNA probe to discriminate single-base mutations was initially confirmed in monoplex-probe real-time PCR experiments. Plasmid DNA samples, including the wild-type strains and 23 common types of *rpoB* sequences with single-base mutations were prepared as template. The results showed that the 23 types of mutant genotypes could be clearly differentiated from the wild targets on the basis of the amplification curves; these mutant genotypes were detected through “probe dropout”. The Cq values of the mutant sequences were determined as negative (Cq = 0) by the second derivative algorithms described in the Materials and methods. The results showed the strong capability of the LNA probe to identify single-base mutations.

All of the LNA probes could combine with the wild-type sequences and generate positive Cq values. In addition, the Cq values of the same concentration of wild DNA (from 7.5 copies/tube to 7.5 × 10^6^ copies/tube) did not significantly vary, although the six probes were aimed at different regions and emitted signals in different fluorescent channels (S1 Fig). The results demonstrated consistent amplification efficiency (determined in accordance with the MIQE Guidelines[26]) and quantifications in the six single-probe PCRs, which facilitate the optimization of the following assay with multiple probes.

### Sensitivity of the multiple-probe assay

**Fig 2:**
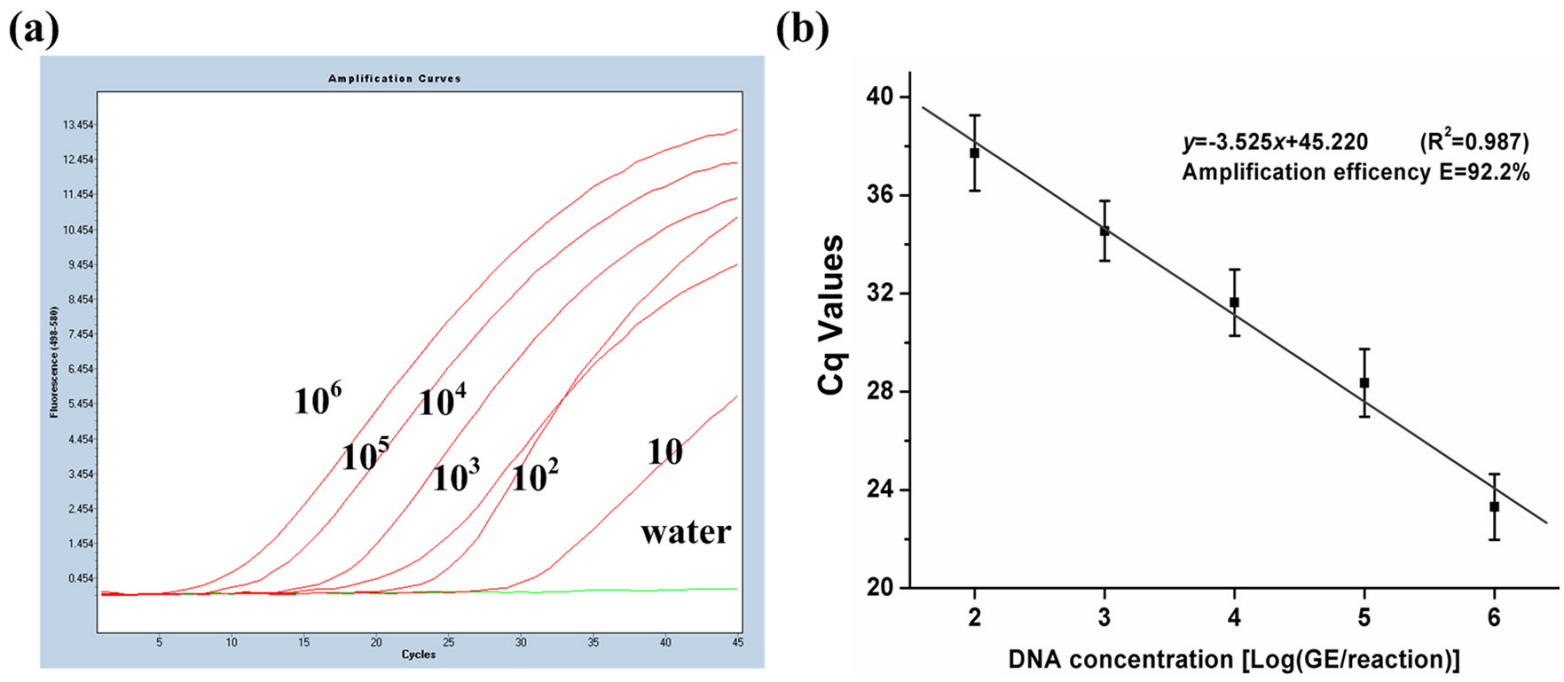
Sensitivity of the probe assay for the detection of *M*. *tuberculosis* H37Rv. (a) Amplification curves of *M*. *tuberculosis* (10 GE/tube to 1.0 × 10^6^ GE/tube) through the multiple-probe real-time PCR. The LOD could be increased to 10 GE/tube through the combination of nested PCR amplification. (b) Quantification curve for *M*. *tuberculosis*. The curve displayed good linearity (R^2^ = 0.987) for *M*. *tuberculosis* at a range of 1.0 × 10^2^ GE/tube to 1.0 × 10^6^ GE/tube.

The real-time PCR assay with multiple LNA probes was developed and optimized after the capacity of each LNA probe was assessed. The genomic DNA was extracted from *M*. *tuberculosis* H37Rv and diluted in series (10 GE/tube to 1.0 × 10^6^ GE/tube) to determine the sensitivity of the assay. The results indicated that the limit of detection (LOD) could reach 100 GE/tube with an average Cq value of 37.72 (S4 Table). The LOD was approximately tenfold higher than that of the real-time PCR with single LNA probe (7.5 copies/tube). The decrease in PCR vitality was caused by two factors. One of the factors involved the presence of the inhibitors from the excess by-products because of the multiple-labeled probes, which may inhibit the activity of the DNA polymerase. The other factor included the competition for the IAC amplification in the same reaction cannot be ignored, especially when the primary *rpoB* target was at a low concentration. The nested PCR amplification was introduced to the real-time PCR assay to overcome these problems; as a result, the LOD was increased to 10 GE/tube (Fig 2a).

The quantification curve was also established to quantitatively detect *M*. *tuberculosis* (Fig 2b). The *x*-axis represents the DNA concentration, and the *y*-axis represents the Cq values (average of the six channels’). The curve displayed good linearity (R^2^ = 0.987) for *M*. *tuberculosis* at a range of 1.0 × 10^2^ GE/tube to 1.0 × 10^6^ GE/tube. The quantification equation was expressed by *y* = −3.525*x* + 52.270. The equation revealed an amplification efficiency of 92.2% for the assay; thus, the multiple-probe PCR was strongly reliable.

### Detection of the *rpoB* mutations associated with RFP resistance

The capability of the assay to discriminate *rpoB* mutations associated with RFP resistance was evaluated to detect RFP-resistant *M*. *tuberculosis*. As shown in Table 1, the 23 common *rpoB* genotypes with single-base mutations could be correctly detected through the assay. The absence of any single-base mutation in the DNA caused the “dropout” of the corresponding LNA probe during amplification (Cq = 0); by contrast, the other LNA probes worked well in the reaction. Our results further indicated that the RFP-resistant *M*. *tuberculosis* can be identified on the basis of the “probe dropout” instead of ΔCq values that is used in most other NAAT assays [14, 15]. Previous studies demonstrated the *rpoB* mutations associated with RFP resistance are mainly single- or double-codon mutations, and triple or more codon mutations have been rarely reported. Therefore, RFP-resistant *M*. *tuberculosis* could be determined as positive if one- or two-probe dropout from the target DNA.

### Specificity of the assay

**Fig 3:**
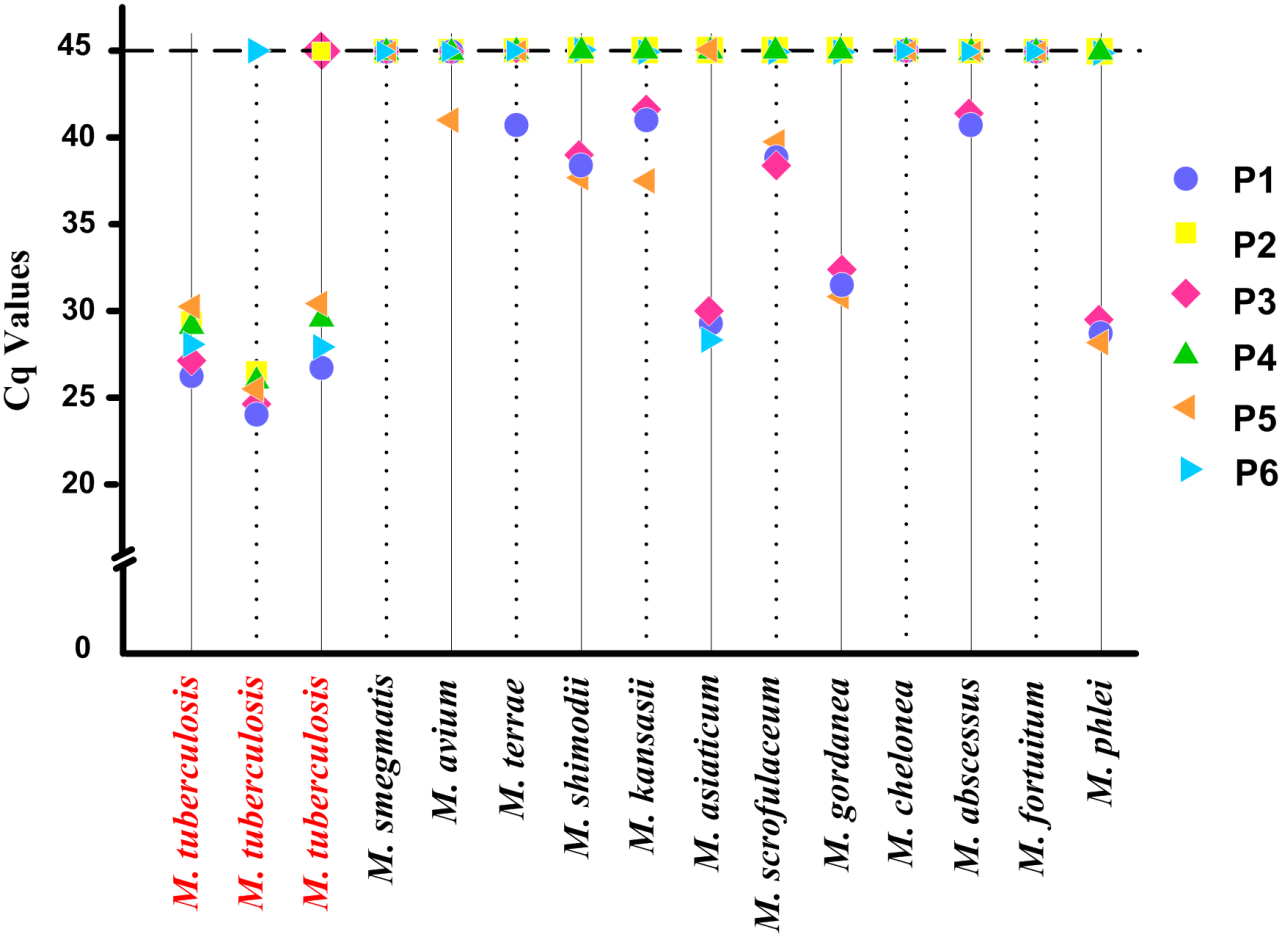
Assay performance with *M*. *tuberculosis* and non-tuberculosis mycobacteria (NTM). *M*. *tuberculosis* was diagnosed with no probe dropouts (RFP-susceptible *M*. *tuberculosis*) or no more than two dropouts (RFP-resistant *M*. *tuberculosis*). The 12 NTM DNA samples were identified with three or more LNA probe dropouts.

The specificity of the assay to *M*. *tuberculosis* was confirmed by evaluating the DNA samples extracted from 12 NTM strains (Fig 3). As the results show, *M*. *tuberculosis* did not have probe dropouts (RFP-susceptible *M*. *tuberculosis*) or did not have more than two dropouts (RFP-resistant *M*. *tuberculosis*). However, three or more LNA probe dropouts occurred when the 12 NTM DNA samples were evaluated (S5 Table). Various degrees of homology were observed between NTM and *M*. *tuberculosis* H37Rv by analyzing the *rpoB* gene sequences. As a result, positive LNA probe signals in amplification of the NTM were observed. However, none of the 12 NTMs were identified as *M*. *tuberculosis* on the basis of the assay results (details in the Materials and methods). Therefore, the assay was sufficiently specific for *M*. *tuberculosis*.

### Validation of 154 clinical isolates through a blind test

A set of 154 clinical isolates with well-defined information on drug susceptibility and *rpoB* gene sequence, including NTM strains, RFP-susceptible *M*. *tuberculosis* strains and RFP-resistant *M*. *tuberculosis*, were used to evaluate the assay performance through a blind test. As the results show (Table 2), 12/12 (100%) of the NTM samples were correctly identified as free of *M*. *tuberculosis*. The remaining isolates were all recognized as *M*. *tuberculosis* by the assay with a high accuracy of 142/142 (100%). Furthermore, of the 142 strains identified as *M*. *tuberculosis*, 88/88 (100%) RFP-susceptible *M*. *tuberculosis* strains were accurately detected through the assay. In addition, 52/54 (96.3%) of the RFP-resistant *M*. *tuberculosis* strains were correctly determined. Of the total samples, only two RFP-resistant strains were falsely diagnosed as RFP-susceptible *M*. *tuberculosis* by the assay. Corresponding DNA Sequences of the RRDR was analyzed in the two discordant samples. An *rpoB* mutation at codon 510 (CAG-CAC) and a mutation codon 522 (TCG-ATG) were then found in the two RFP-resistant samples. However, both mutations were related to low level of resistance to RFP [27, 28] and were not in the panel of the 23 common mutations. Overall, 152/154 clinical isolates (98.7%) were correctly identified by the assay. The whole results showed a highly specific performance of the assay to detect *M*. *tuberculosis* and RFP-resistant *M*. *tuberculosis*.

**Table 2:** Assay performance with 154 clinical isolates.

Sample types	Amount of samples	Amount of samples detected	Amount of samples undetected
**not *M*. *tuberculosis***	12	12 (100%)	0
**RFP-susceptible *M*. *tuberculosis***	88	88 (100%)	0
**RFP-resistant *M*. *tuberculosis***	54	52 (96.3%)	2
**Total**	154	152 (98.7%)	2

## Discussion

In the study, we developed a LNA probe-based assay to determine the genotype of *rpoB* gene to detect *M*. *tuberculosis* and RFP-resistant *M*. *tuberculosis*. 23 different mutations in the RRDR, including the high-frequency mutations at codon 516, 526, and 531, were all identified by the LNA probes and the proposed assay. The assay also showed high specificity to *M*. *tuberculosis* by testing 12 strains of common NTMs. Sensitivity of the detection could reach 10 GE/reaction by further introducing a nested PCR method. Through the evaluation of clinical isolates, the assay presented a high accuracy of 152/154 (98.7%) to detect *M*. *tuberculosis* and RFP-resistant *M*. *tuberculosis*.

The proposed assay utilized six well-designed LNA probes as the genotype detector of RRDR. As a result, all the 23 common mutations were highly distinguished by the LNA probes in a manner of “probe dropout”, which could be easily identified through the amplification curves (Cq = 0), without complex determinations or additional Tm analyses. There are several real-time PCR assays developed for the detection of drug-resistance associated mutations by using other kinds of probes, such as the molecular beacon-based Xpert MTB/RFP assay [5], However, the molecular beacon did not identify the mutations at codon 511 (CGG), codon 516 (TAC and GGC), and codon 526 (AAC and CGC) in the way of “probe dropout” [14]. To discriminate these mutations, the assay still needs complex result determinations based on the value of ΔCq between the matched and mismatched hybridization.

The assay contains seven different probes, including six LNA probes and one IAC Taqman probe. Considering the availability of the real-time PCR instrument and easy application of the assay, we developed a real-time PCR assay in a two-tube form instead of one tube. The design requires only a real-time PCR instrument with four fluorescent detecting channels, such as LightCycler 480 II, ABI 7500, SmartCycler, and CFX 96 real-time PCR detecting system. However, the assay could be improved to a single-tube format to exhibit convenience; thus, the assay could be applicable to common instruments. Furthermore, the assay can be transferred to an automatic diagnostic platform with the capability of “sample-in answer out,” similar to the Cepheid GeneXpert system [29], to achieve point-of-care diagnostics of *M*. *tuberculosis*.

In summary, six LNA probes were designed to detect drug resistance-associated mutations in *rpoB*, and these probes have been validated with excellent specificity to detect the common mutations in the RRDR. Moreover, the LNA probe-based real-time assay developed in the study shows sufficient specificity and sensitivity to detect *M*. *tuberculosis* and drug-resistant *M*. *tuberculosis*, which can become a powerful alternative tool to rapidly diagnose drug-resistant tuberculosis. The assay could also be used to detect the mutations involved in the resistance to other anti-tuberculosis drugs, such as isoniazid, ethambutol, and fluoroquinolone, through designing corresponding specific LNA probes, in future studies.

## Supporting Information

S1 Fig Amplification efficiency of the six LNA probes. (TIF)

S1 Table *rpoB* mutations in the RFP-resistant clinical isolates of *M*. *tuberculosis*. (DOCX)

S2 Table Oligonucleotide primers and probes used in this study. (DOCX)

S3 Table Comparison of four different probe combinations by evaluating the wild-type *rpoB* template at the same concentration. (DOCX)

S4 Table Cq values for the detection of *M*. *tuberculosis* H37Rv (10 GE/tube to 1.0 × 10^6^ GE/tube) through the real-time PCR. (DOCX)

S5 Table Detection results of non-tuberculosis mycobacteria (NTM) and *M*. *tuberculosis*. (DOCX)

## References

[1] Hoek KGP, Van Rie A, van Helden PD, Warren RM, Victor TC. Detecting Drug-Resistant Tuberculosis The Importance of Rapid Testing. Mol Diagn Ther. 2011;15(4):189–94. ISI:000295896500001. 10.2165/11593780-000000000-00000 21913741

[2] Wilson ML. Recent advances in the laboratory detection of Mycobacterium tuberculosis complex and drug resistance. Clin Infect Dis. 2011;52(11):1350–5. Epub 2011/05/21. 10.1093/cid/cir146 . 21596676

[3] McNerney R, Maeurer M, Abubakar I, Marais B, Mchugh TD, Ford N, et al Tuberculosis Diagnostics and Biomarkers: Needs, Challenges, Recent Advances, and Opportunities. J Infect Dis. 2012;205:S147–S58. 10.1093/infdis/jir860 ISI:000303329700002. 22496353

[4] Noor KM, Shephard L, Bastian I. Molecular diagnostics for tuberculosis. Pathology. 2015;47(3):250–6. 10.1097/Pat.0000000000000232 ISI:000351005300009. 25719854

[5] Boehme CC, Nabeta P, Hillemann D, Nicol MP, Shenai S, Krapp F, et al Rapid molecular detection of tuberculosis and rifampin resistance. The New England journal of medicine. 2010;363(11):1005–15. Epub 2010/09/10. 10.1056/NEJMoa0907847 20825313 PMC2947799

[6] Palomino JC. Molecular detection, identification and drug resistance detection in Mycobacterium tuberculosis. Fems Immunol Med Mic. 2009;56(2):103–11. 10.1111/j.1574-695X.2009.00555.x ISI:000266838700001. 19416361

[7] McNerney R, Cunningham J, Hepple P, Zumla A. New tuberculosis diagnostics and rollout. Int J Infect Dis. 2015;32:81–6. 10.1016/j.ijid.2015.01.012 ISI:000352401500014. 25809761

[8] Zhang Y, Yew WW. Mechanisms of drug resistance in Mycobacterium tuberculosis. Int J Tuberc Lung D. 2009;13(11):1320–30. ISI:000271883400004. 19861002

[9] Huang H, Jin Q, Ma Y, Chen X, Zhuang Y. Characterization of rpoB mutations in rifampicin-resistant Mycobacterium tuberculosis isolated in China. Tuberculosis (Edinb). 2002;82(2–3):79–83. Epub 2002/10/03. . 12356458 10.1054/tube.2002.0326

[10] Chakravorty S, Kothari H, Aladegbami B, Cho EJ, Lee JS, Roh SS, et al Rapid, High-Throughput Detection of Rifampin Resistance and Heteroresistance in Mycobacterium tuberculosis by Use of Sloppy Molecular Beacon Melting Temperature Coding. Journal of clinical microbiology. 2012;50(7):2194–202. 10.1128/Jcm.00143-12 ISI:000307360800004. 22535987 PMC3405605

[11] Mboowa G, Namaganda C, Ssengooba W. Rifampicin resistance mutations in the 81 bp RRDR of rpoB gene in Mycobacterium tuberculosis clinical isolates using Xpert (R) MTB/RIF in Kampala, Uganda: a retrospective study. Bmc Infect Dis. 2014;14 10.1186/1471-2334-14-481 ISI:000341513800001. 25190040 PMC4164707

[12] de Freitas FAD, Bernardo V, Gomgnimbou MK, Sola C, Siqueira HR, Pereira MAS, et al Multidrug Resistant Mycobacterium tuberculosis: A Retrospective katG and rpoB Mutation Profile Analysis in Isolates from a Reference Center in Brazil. PloS one. 2014;9(8). ARTN e104100 10.1371/journal.pone.0104100 ISI:000341357200068. PMC4122415 25093512

[13] El-Hajj HH, Marras SAE, Tyagi S, Kramer FR, Alland D. Detection of rifampin resistance in Mycobacterium tuberculosis in a single tube with molecular beacons. J Clin Microbiol. 2001;39(11):4131–7. 10.1128/Jcm.39.11.4131-4137.2001 ISI:000171934200050. 11682541 PMC88498

[14] Helb D, Jones M, Story E, Boehme C, Wallace E, Ho K, et al Rapid Detection of Mycobacterium tuberculosis and Rifampin Resistance by Use of On-Demand, Near-Patient Technology. J Clin Microbiol. 2010;48(1):229–37. 10.1128/Jcm.01463-09 ISI:000276151500032. 19864480 PMC2812290

[15] Wada T, Maeda S, Tamaru A, Imai S, Hase A, Kobayashi K. Dual-probe assay for rapid detection of drug-resistant Mycobacterium tuberculosis by real-time PCR. J Clin Microbiol. 2004;42(11):5277–85. 10.1128/Jcm.42.11.5277-5285.2004 ISI:000225149300053. 15528726 PMC525196

[16] Luo T, Jiang LL, Sun WM, Fu G, Mei J, Gao Q. Multiplex Real-Time PCR Melting Curve Assay To Detect Drug-Resistant Mutations of Mycobacterium tuberculosis. Journal of clinical microbiology. 2011;49(9):3132–8. 10.1128/Jcm.02046-10 ISI:000294416000005. 21752982 PMC3165625

[17] Liu QY, Luo T, Li J, Mei J, Gao Q. Triplex real-time PCR melting curve analysis for detecting Mycobacterium tuberculosis mutations associated with resistance to second-line drugs in a single reaction. J Antimicrob Chemoth. 2013;68(5):1097–103. 10.1093/jac/dks509 ISI:000318105100019. PMC3625432 23288402

[18] Chen XY, Wang B, Yang W, Kong FR, Li CY, Sun ZG, et al Rolling Circle Amplification for Direct Detection of rpoB Gene Mutations in Mycobacterium tuberculosis Isolates from Clinical Specimens. Journal of clinical microbiology. 2014;52(5):1540–8. 10.1128/Jcm.00065-14 ISI:000337915700034. 24574296 PMC3993705

[19] Roh SS, Smith LE, Lee JS, Via LE, Barry CE 3rd, Alland D, et al Comparative Evaluation of Sloppy Molecular Beacon and Dual-Labeled Probe Melting Temperature Assays to Identify Mutations in Mycobacterium tuberculosis Resulting in Rifampin, Fluoroquinolone and Aminoglycoside Resistance. PloS one. 2015;10(5):e0126257 Epub 2015/05/06. 10.1371/journal.pone.0126257 25938476 PMC4418795

[20] Lis P, Kumala A, Spalinski M, Rypula K. Novel locked nucleic acid (LNA)-based probe for the rapid identification of Chlamydia suis using real-time PCR. Bmc Vet Res. 2014;10 Artn 225 10.1186/S12917-014-0225-4 ISI:000342167700001. PMC4177436 25249439

[21] Wang Q, Wang XQ, Zhang JH, Song GH. LNA real-time PCR probe quantification of hepatitis B virus DNA. Exp Ther Med. 2012;3(3):503–8. 10.3892/Etm.2011.442 ISI:000300340300023. 22969919 PMC3438541

[22] Salvi S, D'Orso F, Morelli G. Detection and quantification of genetically modified organisms using very short, locked nucleic acid TaqMan probes. J Agr Food Chem. 2008;56(12):4320–7. 10.1021/Jf800149j ISI:000256893200006. 18494480

[23] Johnson MP, Haupt LM, Griffiths LR. Locked nucleic acid (LNA) single nucleotide polymorphism (SNP) genotype analysis and validation using real-time PCR. Nucleic Acids Res. 2004;32(6). ARTN e55 10.1093/nar/gnh046 ISI:000220898500002. PMC390376 15047860

[24] Josefsen MH, Lofstrom C, Sommer HM, Hoorfar J. Diagnostic PCR: Comparative sensitivity of four probe chemistries. Mol Cell Probe. 2009;23(3–4):201–3. 10.1016/j.mcp.2009.02.003 ISI:000266913300012. 19269316

[25] Somerville W, Thibert L, Schwartzman K, Behr MA. Extraction of Mycobacterium tuberculosis DNA: a question of containment. Journal of clinical microbiology. 2005;43(6):2996–7. Epub 2005/06/16. 10.1128/JCM.43.6.2996-2997.2005 15956443 PMC1151963

[26] Bustin SA, Benes V, Garson JA, Hellemans J, Huggett J, Kubista M, et al The MIQE Guidelines: Minimum Information for Publication of Quantitative Real-Time PCR Experiments. Clinical Chemistry. 2009;55(4). 10.1373/clinchem.2008.112797 19246619

[27] Zaczek A, Brzostek A, Augustynowicz-Kopec E, Zwolska Z, Dziadek J. Genetic evaluation of relationship between mutations in rpoB and resistance of Mycobacterium tuberculosis to rifampin. BMC microbiology. 2009;9:10 Epub 2009/01/17. 10.1186/1471-2180-9-10 19146699 PMC2652454

[28] Williams DL, Spring L, Collins L, Miller LP, Heifets LB, Gangadharam PR, et al Contribution of rpoB mutations to development of rifamycin cross-resistance in Mycobacterium tuberculosis. Antimicrobial agents and chemotherapy. 1998;42(7):1853–7. Epub 1998/07/14. 9661035 10.1128/aac.42.7.1853 PMC105697

[29] Theron G, Zijenah L, Chanda D, Clowes P, Rachow A, Lesosky M, et al Feasibility, accuracy, and clinical effect of point-of-care Xpert MTB/RIF testing for tuberculosis in primary-care settings in Africa: a multicentre, randomised, controlled trial. Lancet. 2014;383(9915):424–35. 10.1016/S0140-6736(13)62073-5 ISI:000330673700032. 24176144

