# Correction: Locked Nucleic Acid Probe-Based Real-Time PCR Assay for the Rapid Detection of Rifampin-Resistant *Mycobacterium tuberculosis*

**DOI:** 10.1371/journal.pone.0157275

**Published:** 2016-06-06

**Authors:** Yong Zhao, Guilian Li, Chongyun Sun, Chao Li, Xiaochen Wang, Haican Liu, Pingping Zhang, Xiuqin Zhao, Xinrui Wang, Yi Jiang, Ruifu Yang, Kanglin Wan, Lei Zhou

S2 Table lists incorrect sequences. The authors have provided the correct S2 Table. It can be viewed below.

## Supporting Information

S2 Table Oligonucleotide primers and probes used in this study. (DOCX)
