# Supplementary figures and images for: Locked Nucleic Acid Probe-Based Real-Time PCR Assay for the Rapid Detection of Rifampin-Resistant Mycobacterium tuberculosis
Source: PLoS One. 2015 Nov 24;10(11):e0143444. doi: 10.1371/journal.pone.0143444 (PMC4657947; doi:10.1371/journal.pone.0143444)

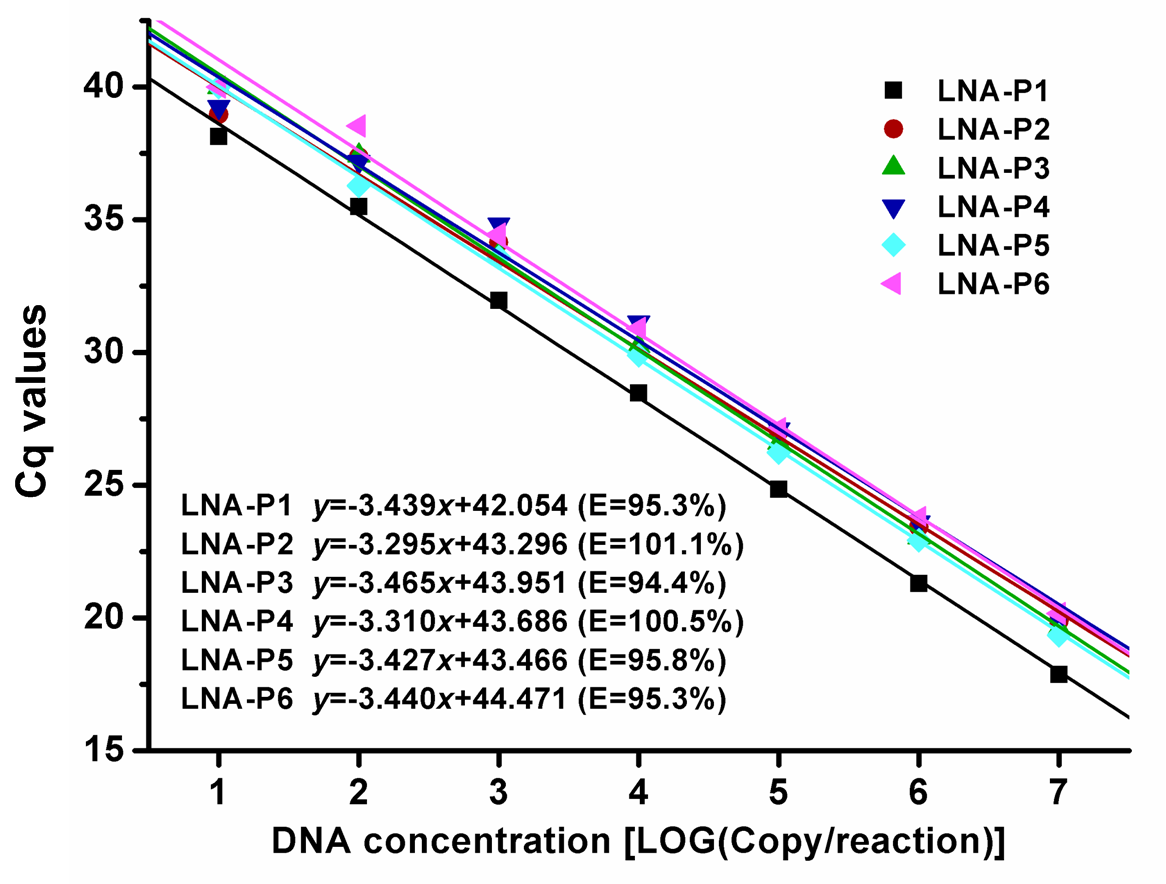

Supplement: S1 Fig — (TIF) [file pone.0143444.s001.tif]
