# Supplementary material for: Locked Nucleic Acid Probe-Based Real-Time PCR Assay for the Rapid Detection of Rifampin-Resistant Mycobacterium tuberculosis
Source: PLoS One. 2015 Nov 24;10(11):e0143444. doi: 10.1371/journal.pone.0143444 (PMC4657947; doi:10.1371/journal.pone.0143444)
Supplement: S1 Table — (DOCX) [file pone.0143444.s002.docx]

**S1 Table. *rpoB* mutations in the RFP-resistant clinical isolates of *M. tuberculosis*.**

| **Mutation codon** | **Mutation type** | **Number of samples** | **Percentage** |
| --- | --- | --- | --- |
| **510** | **CAG-CAT** | **1** | **1.9%** |
| **510** | **CAG-CAC*** | **1** | **1.9%** |
| **511** | **CTG-CCG** | **5** | **9.3%** |
| **513** | **CAA-AAA** | **1** | **1.9%** |
| **516** | **GAC-TAC** | **4** | **7.4%** |
| **516** | **GAC-GTC** | **3** | **5.6%** |
| **516** | **GAC-GGC** | **2** | **3.7%** |
| **522** | **TCG-TTC** | **1** | **1.9%** |
| **522** | **TCG-ATG*** | **1** | **1.9%** |
| **526** | **CAC-TAC** | **6** | **11.1%** |
| **526** | **CAC-AAC** | **5** | **9.3%** |
| **526** | **CAC-GAC** | **3** | **5.6%** |
| **526** | **CAC-TGC** | **3** | **5.6%** |
| **526** | **CAC-CGC** | **3** | **5.6%** |
| **526** | **CAC-CTC** | **1** | **1.9%** |
| **531** | **TCG-TTG** | **15** | **24.1%** |
| **531** | **TCG-TTT** | **1** | **1.9%** |
| **Total** | **-** | **54** | **100%** |

* The two types of mutations are not in the panel of the targeted 23 mutations of the assay.
