# Supplementary material for: Locked Nucleic Acid Probe-Based Real-Time PCR Assay for the Rapid Detection of Rifampin-Resistant Mycobacterium tuberculosis
Source: PLoS One. 2015 Nov 24;10(11):e0143444. doi: 10.1371/journal.pone.0143444 (PMC4657947; doi:10.1371/journal.pone.0143444)
Supplement: S2 Table — (DOCX) [file pone.0143444.s003.docx]

**S2 Table. Oligonucleotide primers and probes used in this study.**

| **Primer or probe** | **Sequence (5′→3′)** |
| --- | --- |
| rpoB-F1 | aggacgtggaggcgatcac |
| rpoB-F2 | tggtcgccgcgatcaag |
| rpoB-R | cacgtcgcggacctccag |
| BgSP-F | cagctgctggacaaggacaattc |
| BgSP-R | cttccacccgaggatgaagt |
| LNA-P1 | Cy5-cggcacC*Agccag-BHQ-3 |
| LNA-P2 | Fam-cagCtgAgCCAattc-BHQ-1 |
| LNA-P3 | Hex-atgGaCCaGaacaac-BHQ-1 |
| LNA-P4 | Cy5-ccgctgtcGgGgttg-BHQ-3 |
| LNA-P5 | Hex-acccacaaGCGCcga-BHQ-1 |
| LNA-P6 | Fam-actgtCggCGCtg-BHQ-1 |
| Taqman-IAC | Rox-gaattcgctagtgaaactgatgctcagcaa-BHQ-2 |

*LNA monomer in the probe is written in capital letters.
