# Supplementary material for: Locked Nucleic Acid Probe-Based Real-Time PCR Assay for the Rapid Detection of Rifampin-Resistant Mycobacterium tuberculosis
Source: PLoS One. 2015 Nov 24;10(11):e0143444. doi: 10.1371/journal.pone.0143444 (PMC4657947; doi:10.1371/journal.pone.0143444)
Supplement: S3 Table — (DOCX) [file pone.0143444.s004.docx]

**S3 Table. Comparison of four different probe combinations by testing wild-type *rpoB* template at the same concentration.** Probe combination 1 was selected in the assay with optimal sensitivity (AVG Cq) and amplification consistency (CV) of the six probes.

| **Probe combinations** | **Probes in**  **Tube A** | **Probes in**  **Tube B** | **Cq values amplified by the six probes** | | | | | | **AVG Cq** | **Max**  **∆Cq** | **CV** |
| --- | --- | --- | --- | --- | --- | --- | --- | --- | --- | --- | --- |
|  |  |  | **P1** | **P2** | **P3** | **P4** | **P5** | **P6** |  |  |  |
| **1** | 1, 3, and 6 | 2, 4, and 5 | 26.13 | 27.19 | 26.75 | 27.91 | 29.09 | 27.88 | 27.49 | 2.96 | 3.8% |
| **2** | 3, 4, and 6 | 1, 2, and 5 | 24.77 | 26.41 | 31.76 | 31.23 | 25.63 | 32.49 | 28.71 | 7.72 | 12.1% |
| **3** | 1, 5, and 6 | 2, 3, and 4 | 24.64 | 29.52 | 30.89 | 30.36 | 25.39 | 27.66 | 28.07 | 6.25 | 9.3% |
| **4** | 4, 5, and 6 | 1, 2, and 3 | 28.82 | 27.42 | 26.55 | 25.84 | 29.78 | 30.53 | 28.15 | 4.69 | 6.6% |
