# Supplementary material for: Locked Nucleic Acid Probe-Based Real-Time PCR Assay for the Rapid Detection of Rifampin-Resistant Mycobacterium tuberculosis
Source: PLoS One. 2015 Nov 24;10(11):e0143444. doi: 10.1371/journal.pone.0143444 (PMC4657947; doi:10.1371/journal.pone.0143444)
Supplement: S4 Table — (DOCX) [file pone.0143444.s005.docx]

**S4 Table. Cq values for the detection of *M. tuberculosis* H37Rv (10 GE/tube to 1.0 × 10^6^ GE/tube)** **through the real-time PCR.**

| **Concentration (GE/reaction)** | **Cq values** | | | | | | **AVG Cq** a | **IAC Cq** b |
| --- | --- | --- | --- | --- | --- | --- | --- | --- |
|  | **P1** | **P2** | **P3** | **P4** | **P5** | **P6** |  |  |
| **10** | 0 | 0 | 0 | 0 | 0 | 0 | 0 | 34.07±0.32 |
| **1.0 × 10^2^** | 36.51±0.23 | 37.10±0.64 | 36.46±0.35 | 38.37±0.84 | 40.51±0.61 | 37.40±0.33 | 37.72±1.53 | 34.55±0.29 |
| **1.0 × 10^3^** | 33.50±0.37 | 34.06±0.53 | 33.38±0.51 | 35.07±0.53 | 36.65±0.50 | 34.68±0.33 | 34.55±1.22 | 34.34±0.33 |
| **1.0 × 10^4^** | 30.30±0.13 | 31.33±0.03 | 30.56±0.08 | 31.78±0.06 | 34.10±0.14 | 31.71±0.05 | 31.63±1.35 | 34.60±0.40 |
| **1.0 × 10^5^** | 26.94±0.03 | 28.42±0.04 | 27.25±0.07 | 28.56±0.03 | 30.84±0.08 | 28.17±0.06 | 28.36±1.38 | 35.91±1.10 |
| **1.0 × 10^6^** | 21.89±0.04 | 23.44±0.12 | 22.15±0.08 | 23.55±0.04 | 25.65±0.11 | 23.18±0.11 | 23.31±1.34 | 39.89±1.09 |

a AVG Cq was derived from the average Cq values corresponding to the six LNA probes.

b IAC DNA template of the reactions kept the same concentration of 2500 GE/tube.
