# Supplementary material for: Locked Nucleic Acid Probe-Based Real-Time PCR Assay for the Rapid Detection of Rifampin-Resistant Mycobacterium tuberculosis
Source: PLoS One. 2015 Nov 24;10(11):e0143444. doi: 10.1371/journal.pone.0143444 (PMC4657947; doi:10.1371/journal.pone.0143444)
Supplement: S5 Table — (DOCX) [file pone.0143444.s006.docx]

**S5 Table. Detection results of non-tuberculosis mycobacteria (NTM) and *M. tuberculosis*.**

| **NTM** | **Cq values** | | | | | |
| --- | --- | --- | --- | --- | --- | --- |
|  | **LNA-P1** | **LNA-P2** | **LNA-P3** | **LNA-P4** | **LNA-P5** | **LNA-P6** |
| ***M. avium*** | 0 | 0 | 0 | 0 | 41.7 | 0 |
| ***M. terrae*** | 40.74 | 0 | 0 | 0 | 0 | 0 |
| ***M. shimodii*** | 37.94 | 0 | 37.69 | 0 | 37.08 | 0 |
| ***M. kansasii*** | 41.76 | 0 | 41.69 | 0 | 37.01 | 0 |
| ***M. asiaticum*** | 29.21 | 0 | 29.65 | 0 | 0 | 29.77 |
| ***M. scrofulaceum*** | 39.13 | 0 | 39.1 | 0 | 39.04 | 0 |
| ***M. gordanea*** | 33.53 | 0 | 33.56 | 0 | 31.2 | 0 |
| ***M. chelonea*** | 0 | 0 | 45.57 | 0 | 0 | 0 |
| ***M. abscessus*** | 40.11 | 0 | 40.29 | 0 | 0 | 0 |
| ***M. fortuitum*** | 0 | 0 | 0 | 0 | 46.02 | 0 |
| ***M. phlei*** | 27.85 | 0 | 28.18 | 0 | 27.74 | 0 |
| ***M. smegmatis*** | 0 | 0 | 0 | 0 | 0 | 0 |
| **RFP-susceptible *M. tuberculosis*** | 26.73 | 29.01 | 27.21 | 29.05 | 30.15 | 27.54 |
| **RFP-resistant *M. tuberculosis*** | 23.95 | 26.14 | 24.35 | 26.2 | 26.63 | 0 |
| **RFP-resistant *M. tuberculosis*** | 25.93 | 0 | 0 | 29.28 | 30.58 | 27.44 |
