# Supplementary material for: Correction: Locked Nucleic Acid Probe-Based Real-Time PCR Assay for the Rapid Detection of Rifampin-Resistant Mycobacterium tuberculosis
Source: PLoS One. 2016 Jun 6;11(6):e0157275. doi: 10.1371/journal.pone.0157275 (PMC4894557; doi:10.1371/journal.pone.0157275)
Supplement: S2 Table — (DOCX) [file pone.0157275.s001.docx]

**S2 Table. Oligonucleotide primers and probes used in this study.**

| **Primer or probe** | **Sequence (5′→3′)** |
| --- | --- |
| rpoB-F1 | cgtggaggcgatcacacc |
| rpoB-F2 | tcgccgcgatcaaggagt |
| rpoB-R | cacgtcgcggacctccag |
| BgSP-F | cagctgctggacaaggacaa |
| BgSP-R | cttccacccgaggatgaagt |
| LNA-P1 | Cy5-ttcggcaCCAgcc-BHQ-3 |
| LNA-P2 | Fam-tgaattGGCtCagCt-BHQ-1 |
| LNA-P3 | Hex-catGGaCCAgaaca-BHQ-1 |
| LNA-P4 | Cy5-tcaACCccgacagcg-BHQ-3 |
| LNA-P5 | Hex-cccacaagCGCCg-BHQ-1 |
| LNA-P6 | Fam-cgccgacAgtcg-MGB |
| Taqman-IAC | Rox-ttcgctagtgaaactgatgctcagcaag-BHQ-2 |

*LNA monomer in the probe is written in capital letters.
